# Alpha-Linolenic Acid Exerts an Endothelial Protective Effect against High Glucose Injury via PI3K/Akt Pathway

**DOI:** 10.1371/journal.pone.0068489

**Published:** 2013-07-05

**Authors:** Wei Zhang, Rong Li, Jia Li, Wenqing Wang, Ru Tie, Fei Tian, Xiangyan Liang, Wenjuan Xing, Yong He, Liang Yu, Miaomiao Xi, Siwang Wang, Qiangsun Zheng, Haifeng Zhang

**Affiliations:** 1 Department of Cardiology, Tangdu hospital, Fourth Military Medical University, Xi'an, China; 2 Experiment Teaching Center, Fourth Military Medical University, Xi'an, China; 3 Department of Geratology, Xijing Hospital, Fourth Military Medical University, Xi'an, China; 4 Department of Physiology, Fourth Military Medical University, Xi'an, China; 5 Department of Hematology, Tangdu Hospital, Fourth Military Medical University, Xi'an, China; 6 Department Pharmacy, Xijing Hospital, Fourth Military Medical University, Xi'an, China; 7 Institute of Pharmaceutical Research, Fourth Military Medical University, Xi'an, China; Thomas Jefferson University, United States of America

## Abstract

Mounting evidence has indicated that the cardiovascular protective effects of dietary alpha-linolenic acid (ALA), but whether ALA exerts an endothelial protective effect against high glucose injury and the underlying mechanisms remain largely unknown. Streptozocin-induced diabetic rats were randomized treated orally for 4 weeks with vehicle (0.01% alcohol) or ALA (500 µg/kg per day by gavage). Human umbilical vein endothelial cells (HUVECs) were exposed to high glucose (28 mmol/L) stimulation for 48 hours. ALA significantly improved concentration-dependent vasorelaxation to ACh in diabetic aortic segments and inhibited endothelial inflammation as evidenced by decreased soluble P-selectin and intercellular adhesion molecule-1 (ICAM-1) in diabetic rats. Furthermore, both P-selectin and ICAM-1 expression were increased significantly in high glucose-induced HUVECs, resulting in enhanced neutrophils adhesion to HUVECs compared with normal glucose group. Treatment with ALA (50 µmol/L) increased Akt phosphorylation, attenuated P-selectin and ICAM-1 expressions and thus inhibited neutrophils adhesion in HUVECs exposed to high glucose, all of which was blocked by the PI3K inhibitors LY294002 and wortmannin. These data indicates that ALA inhibits endothelial inflammation and improved endothelial function in STZ-induced diabetic rats. The anti-adhesive effect of ALA against high glucose injury may partially be mediated by the PI3K/Akt pathway.

## Introduction

Cardiovascular disease is one of the leading causes of death in the western world and diabetes mellitus has been identified as a primary risk factor [1]. Endothelial inflammation and dysfunction play important roles in the pathogenesis of diabetic vascular complications [2], [3]. One of the key initial events in that pathological process is the adhesion of neutrophils to endothelial cells which is largely mediated by cellular adhesion molecules (CAMs), such as intercellular adhesion molecule-1 (ICAM-1), P-selectin and E-selectin. During endothelial inflammation/activation, soluble forms of these molecules are released from shedding or proteolytic cleavage from the endothelial cell surface and may reflect overexpression of their respective membrane-bound forms [4]. Elevated plasma levels of endothelial cell adhesion molecules have also been documented in diabetic patients [4], [5]. It is, therefore, thought that prevention of high glucose-mediated endothelial CAMs expression and restoration of endothelial function may have important implications for pharmacological attempts to prevent the development of vascular diseases occurring in diabetes.

Evidence from both epidemiologic studies and clinical trials demonstrate substantial cardiovascular protective effects of n-3 polyunsaturated fatty acids (PUFAs) [6], [7]. However, marine-derived n-3 fatty acids such as eicosapentaenoic acid (EPA) and docosahexanoeic acid (DHA) are not as widely available as plant-derived n-3 PUFA, alpha-linolenic acid (ALA), because of the cost and supply constraints of seafood compared with plant sources. And, it should be emphasized that fish oil may not fully reproduce the effects of ALA [8]. Therefore, the effect of ALA on cardiovascular diseases risk is therefore of considerable public health importance, particularly for populations with low consumption or availability of fatty fish. Epidemiologic data have shown beneficial effects of dietary ALA on risk of coronary heart diseases [9] and reduced calcified atherosclerotic plaque in coronary arteries [10]. Dietary fats rich in ALA have been reported to modulate the inflammatory response in dyslipidemia patients [11]. Increased intakes of dietary ALA elicit anti-inflammatory effects by inhibiting inflammatory cytokines production in peripheral blood mononuclear cells [12]. Therefore, ALA appears to decrease cardiovascular disease risk by inhibiting inflammatory response beyond its lipid-lowering effects. However, little is known about the effects of ALA on endothelial inflammation and dysfunction under high glucose condition.

By activating the downstream serine/threonine kinase Akt, phosphatidylinositol 3′- kinase (PI3K) plays an important role in cellular proliferation and survival. It is reported that PI3K/Akt pathway also participates in the cellular inflammatory response [13], [14]. Previous study showed that EPA and DHA attenuated ox-LDL-induced expression of adhesion molecules in human coronary artery endothelial cells by modulation of Akt activation [15]. It is reported that high glucose decreases Akt phosphorylation and activation [16], [17].

Therefore, the present study was designed to determine whether ALA may inhibit endothelial inflammation and improve endothelial function in diabetic rats; and if so, to investigate the role of PI3K/Akt pathway in the anti-inflammatory effect of ALA on endothelial cells against high glucose injury.

## Materials and Methods

### Ethics Statement

The experiments were performed in adherence with the National Institutes of Health Guidelines for the Use of Laboratory Animals. All experiments involving rats were reviewed and approved by the Ethics Committee for animal care and use of Fourth Military Medical University, P.R. China. The use of human umbilical vein endothelial cell lines (HUVEC) was reviewed and approved by Ethics Committee of Xijing Hospital, Fourth Military Medical University, P.R. China, and written informed consent was given by persons donating umbilical cords for use of this sample in research as described in our previous study [18].

### Animals and Induction of Diabetes

Male Sprague-Dawley rats were housed in temperature controlled cages (20–22°C) with a 12-hour light-dark cycle, and given free access to water and formulated diets. The animals were acclimatized for a 2-week period before starting the protocol. A single dose streptozotocin (STZ, Sigma) regimen was used to induce pancreatic-islet-cell destruction and persistent hyperglycemia. STZ was freshly dissolved in sterile sodium citrate buffer (25 mmol/L, pH 4.5) and used within 10 minutes. Rats received a single 60 mg/kg intravenous injection of STZ (diabetic model group) or citrate buffer (control group). Diabetes was identified 72 hours after injection by blood glucose higher than 11 mmol/L [19]. Immediately after diabetes induction, different groups of rats were treated orally for 4 weeks with vehicle (0.01% alcohol) or ALA (500 µg/kg per day by gavage). ALA was kindly provided by Institute of Pharmaceutical Research of Fourth Military Medical University, Xi’an, China, with purity>91.6%. After 4 weeks of treatment, fasting blood glucose and plasma insulin concentration were measured by a glucosemeter (Lifescan Inc) and a commercial radioimmunoassay kit (Beijing North Institute of Biological Technology), respectively. The descending thoracic aorta was isolated for determination of endothelial activation and function.

### Determination of Endothelial Activation and Function

Endothelial function was determined by comparing the vasorelaxation response to acetylcholine (ACh), an endothelium-dependent vasodilator, with that of NaNO_2_, an endothelium-independent vasodilator. Briefly, the aorta was cleaned of connective tissue and cut into 3 mm rings. Rings were mounted onto hooks, suspended in organ chambers filled with Krebs buffer and aerated with 95% O_2_ and 5% CO_2_ at 37°C, and connected to force transducers (WPI, Sarasota, FL) to record changes via a Maclab data acquisition system. After equilibration for 1 hour at a preload of 1 g, the rings were precontracted with norepinephrine (NE, 0.1 nM). Once a stable contraction was achieved, the rings were exposed to cumulative concentrations of ACh (10^−11^ to 10^−5^ mol/L). After the cumulative response stabilized, the rings were washed and allowed to equilibrate to baseline. The procedure was then repeated with an endothelium independent vasodilator (NaNO_2_, 10^−8^ to 10^−5^ mol/L) to determine smooth muscle function and sensitivity to NO. Endothelial dysfunction was defined as a reduced vasorelaxation in response to ACh with a normal response to NaNO_2_.

Endothelial activation was assessed by measuring endothelial soluble adhesion molecules in the blood [4], [20]. Soluble P-selectin (sP-selectin) and soluble ICAM-1 (sICAM-1) were measured using quantitative sandwich enzyme immunoassay kits (R&D Systems) following protocols provided by the manufacturer.

### Cell Culture and Treatment

HUVECs were harvested from freshly discarded human umbilical cords by collagenase perfusion as described previously [14], [21]. The cells were grown in endothelial growth medium supplemented with bovine brain extract in 5% CO_2_. Cells were used within passage 4 after primary culture. Human polymorphonuclear leukocytes (PMN) were isolated from the venous blood of healthy adults using standard dextran sedimentation and gradient separation on Histopaque-1077 (Sigma). PMN suspensions prepared with this technique were ≥95% pure. These procedures were approved by our institutional review board. Each subject provided a written consent and was compensated for participating in the study. Then endothelial cells were incubated with high glucose (28 mmol/L, HG group) for 48 hours in the presence or absence of ALA. Parallel groups were pretreated with different concentrations of ALA (10, 50, 100, 200 µmol/L) for 18 hours before incubation with high glucose. ALA was dissolved in a NaOH/BSA complex solution as previously described [22]. There were no detectable differences in PMN adherence among treatment groups with different NaOH/BSA concentrations used in the present study with both normal and high glucose concentrations (data not shown). To further examine the role of PI3K/Akt pathway in the effect of ALA, another two groups of HUVECs were pretreated with the specific PI3K inhibitors LY294002 (10 µmol/L; Sigma) or wortmannin (100 nmol/L; Sigma) before ALA (50 µmol/L) was added. Additionally, cells cultured in normal glucose (5.5 mmol/L) served as normal control group (NG group). Our preliminary data showed that ALA did not affect neutrophils adherence or CAMs expression in normal glucose media. Neither the specific PI3K inhibitor wortmannin nor LY 294002 had effects on neutrophils adherence and CAMs expression in normal glucose media (data not shown).

### Neutrophil-endothelial Cell Adhesion Assay

The neutrophil-endothelial adhesion assay was reported previously [23]. Briefly, HUVECs at 1×10^7^ cells/well were seeded into 48-well collagen I-coated plates and incubated for 48 hours at 37°C in the presence or absence of various agents. After treatment, the culture medium was replaced with Hank’s balanced salt solution (HBSS; Gibco), and human PMNs were added at a concentration of 1×10^5^ cells/well and allowed to adhere for 30 minutes at 37°C. The monolayers were gently washed twice with 500 µL of warmed HBSS to wash out non-adhered neutrophils. Then, myeloperoxidase (MPO) activity was used as an index of neutrophil accumulation as determined by measuring the H_2_O_2_-dependent oxidation of 3,3′,5,5′-tetramethylbenzidine (Sigma). Values were calculated from a purified leukocyte MPO standard (Sigma). Duplicate samples were diluted 1∶2 with 50 mM phosphate buffer (pH 6.0) containing 0.5% HTAB and incubated with 0.1 ml of ‘K-Blue' substrate (stabilized H_2_O_2_ and TMB) for 10 min. The plate was shaken for 5 s and absorbance measured at 620 nm. MPO activity was expressed as MPO U site^−1^. PMN adherence was expressed as the ratio of MPO activity of adhered neutrophils to that of the total neutrophils added (1×10^5^ cells).

### Assay of Surface Expressions of CAMs

Surface expressions of CAMs (ICAM-1 and P-selectin) were measured as reported previously [23]. Briefly, after treatment of cells with various agents for 48 hours in collagen I-coated 24-well plates, cells were fixed for 10 minutes at room temperature with 1% paraformaldehyde in PBS. After three washes with 1 ml HBSS/PBS (1∶1), cells were incubated for 1 hour at 37°C with an anti-adhesion molecule antibody (primary antibody; diluted 1∶250–500; Sigma) in HBSS/PBS containing 5% fetal calf serum (FCS). Cells were washed twice with 0.5 ml HBSS/PBS and incubated for 1 hour at 37°C with horseradish peroxidase-conjugated goat anti-mouse IgG (diluted 1∶5000; Sigma) in HBSS/PBS containing 5% FCS. Cells were then washed three times with 0.5 ml of HBSS/PBS and incubated for 1 hour at 37°C in the dark with 0.25 ml of 0.003% H_2_O_2_ plus 0.1 mg/ml 3,3′,5,5′-tetramethylbenzidine as a substrate. The color reaction was stopped by adding 75 µl of 8 N H_2_SO_4_, and the samples were transferred to 96-well plates, which were read at 450 nm on a plate reader. A control consisted of cells stained only with the secondary antibody.

### Western Blot Assay

Proteins were extracted from HUVECs and the immunoblots were probed with primary antibodies for ICAM-1, P-selectin, phosphorylated Akt (Ser-473) overnight at 4°C followed by incubation with the corresponding secondary antibodies at room temperature for 1 hour. The blots were visualized with ECL-plus reagent. Phosphorylated Akt immunoblots were then stripped with strip buffer at 50°C for 30 min and reblotted for total Akt.

### Statistical Analysis

All values were presented as mean ± SEM. All data were analyzed using ANOVA followed by the Bonferroni correction for post hoc *t* test. P<0.05 was considered to be statistically significant.

## Results

### Blood Glucose and Plasma Insulin Concentrations in STZ-induced Diabetic Rats

Compared with the control group, blood glucose increased significantly in diabetic rats (28.6±1.6 mmol/L *vs.* 6.7±0.6 mmol/L in Control group, *P*<0.01). Moreover, STZ administration resulted in a significant reduction in plasma insulin concentration of diabetic rats (9.2±0.5 µU/mL *vs.* 68.2±6.2 µU/mL in Control group, *P*<0.01). Treatment of diabetic rats with 500 µg/kg ALA did not have any significant effect on blood glucose (26.5±1.8 mmol/L) and plasma insulin (9.6±0.2 µU/mL) concentrations compared with vehicle-treated group (both *P*>0.05).

### ALA Improved Vasular Dysfunction in STZ-induced Diabetic Rats

**Figure 1:**
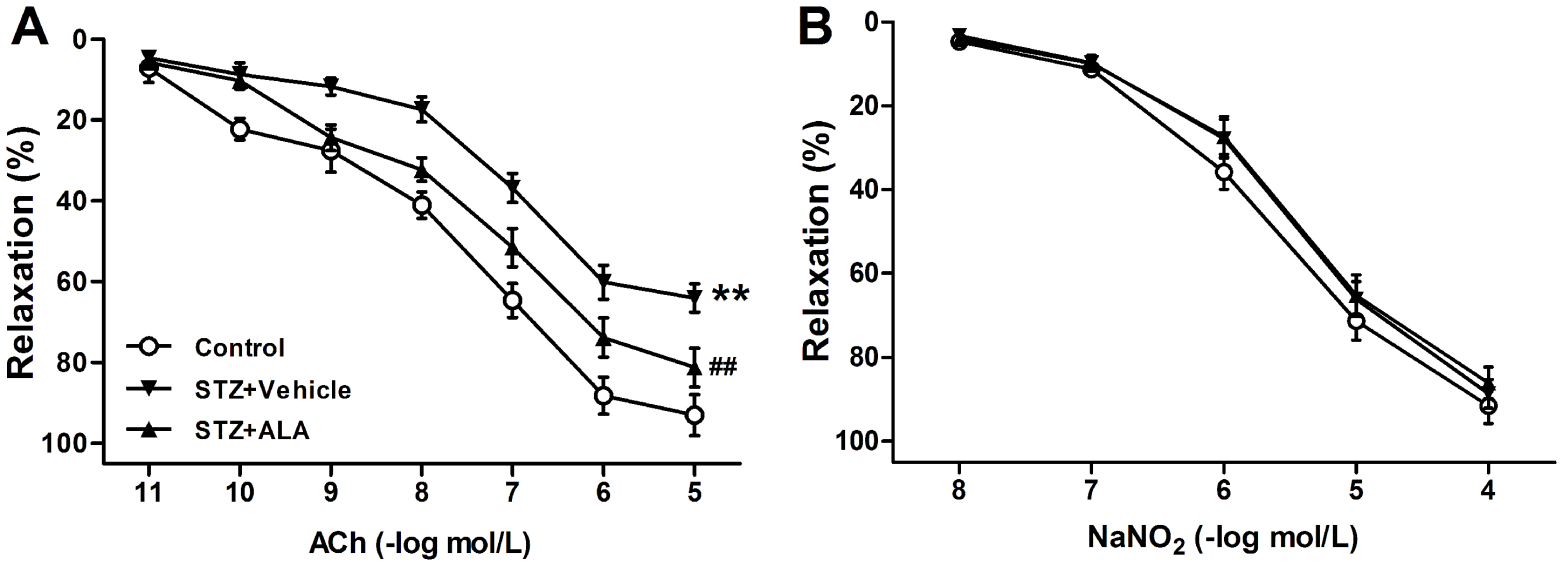
Vasorelaxation to ACh (A) or NaNO_2_ (B) in aortic segments from control and diabetic rats. STZ+Vehicle: diabetic rats treated with vehicle; STZ+ALA: diabetic rats treated with alpha-linolenic acid (ALA). Values are mean±SEM. n = 8–10 vascular segments/group from 8 rats. **P<0.01 *vs.* control. ^##^ *P*<0.01 *vs.* STZ+Vehicle.

Consistent with previously reported results [24], concentration-dependent vasorelaxation in response to ACh was impaired in vascular segments isolated from STZ-induced diabetic rats (64.1% ±3.5% *vs.* 93.1% ±5.1% to 10^−5^ mol/L ACh, n = 8–10 vascular segments/group from 8 rats, *P*<0.01 *vs.* control.) (Figure 1A). However, concentration-dependent vasorelaxation in response to NaNO_2_, an endothelium-independent vasodilator, remained unchanged in these vessels (*P*>0.05) (Figure 1B). ALA treatment significantly increased the ACh-induced vasorelaxation (81.3% ±4.8% to 10^−5^ mol/L ACh, *P*<0.01 *vs.* STZ+Vehicle.) but not influenced vascular response to NaNO_2_ in diabetic rats (Figure 1). These results indicate that STZ-induced diabetes caused significant endothelial dysfunction and treatment with ALA alleviated endothelial dysfunction as evidenced by a significant improvement of dose-response curve to ACh in diabetic rats.

### ALA Decreased Soluble Cellular Adhesion Molecules Expression in STZ-induced Diabetic Rats

**Figure 2:**
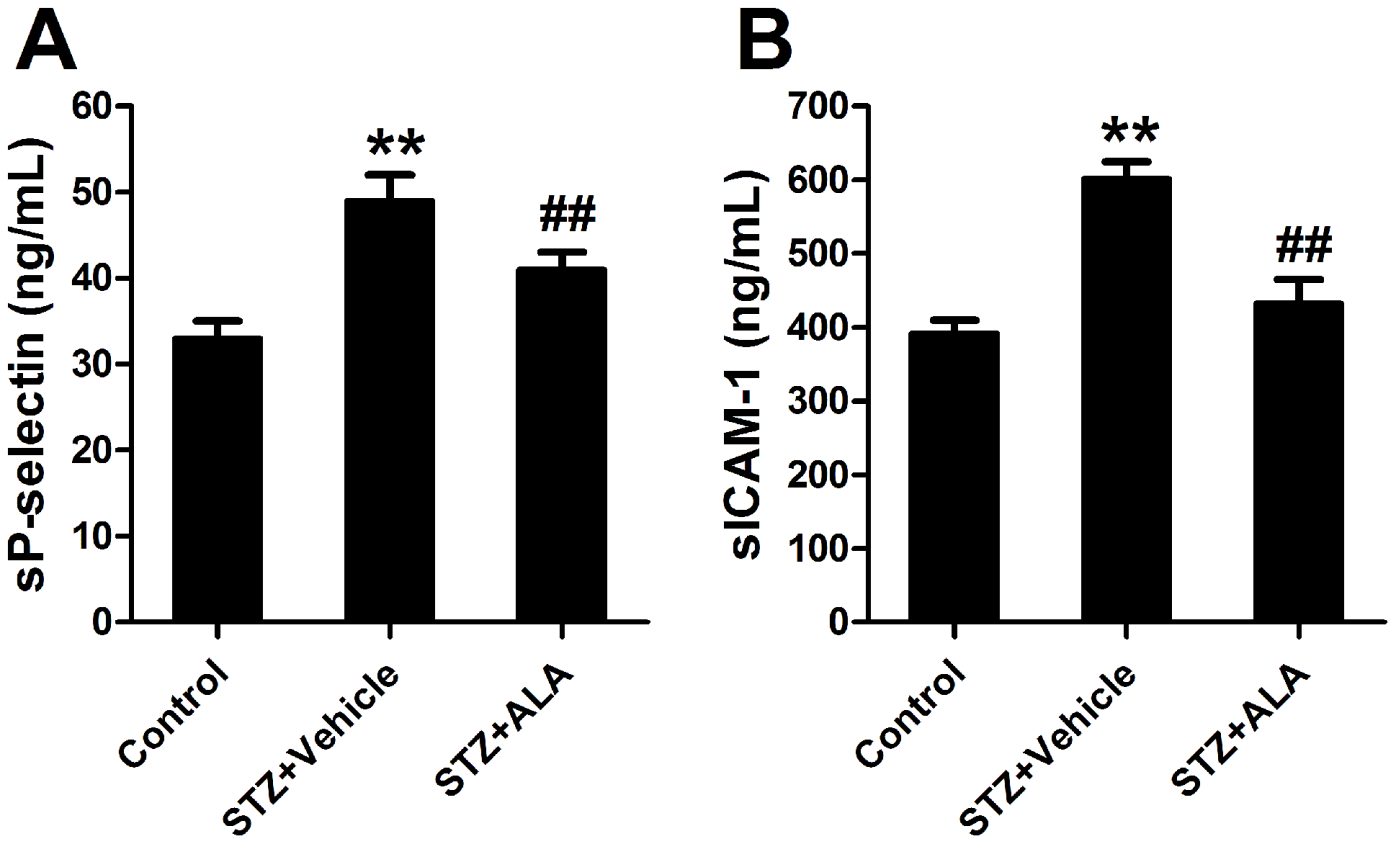
Plasma levels of soluble P-selectin (A) and ICAM-1 (B) in control and diabetic rats. STZ+Vehicle: diabetic rats treated with vehicle; STZ+ALA: diabetic rats treated with ALA. Values are mean±SEM. n = 8. **P<0.01 *vs.* control. ^##^ *P*<0.01 *vs.* STZ+Vehicle.

Endothelial inflammation/activation was assessed by measuring endothelial soluble adhesion molecules in the plasma. STZ-induced diabetes rats had significantly higher levels of sP-selectin (49±3 ng/mL *vs.* 33±2 ng/mL in control group, n = 8, *P<*0.01) and sICAM-1 (602±23 ng/mL *vs.* 392±18 ng/mL, *P<*0.01) compared with control group (Figure 2). Treatment with ALA decreased both sP-selectin (41±2 ng/mL, *P<*0.01) and sICAM-1 (433±32 ng/mL, *P<*0.01) in STZ-induced diabetic rats compared with the vehicle group. Together with our data about vascular function, these results demonstrated that ALA inhibited endothelial activation and improved endothelial function in STZ-induced diabetic rats, indicating the anti-adhesive and endothelial protective effects of ALA in diabetes.

### ALA Inhibited High Glucose-induced Endothelial-neutrophil Adhesion

**Figure 3:**
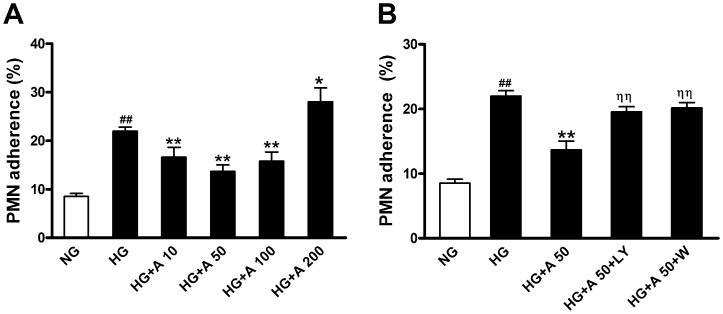
PMN adherence to human umbilical vein endothelial cells (HUVECs) exposed to high glucose. A: HUVECs exposed to high glucose (28 mmol/L) for 48 h were pretreated with different concentrations (10, 50, 100, 200 µmol/L) of ALA. B: high glucose-stimulated HUVECs were treated with ALA (50 µmol/L) with different PI3K inhibitors. NG: normal glucose condition (5.5 mmol/L); HG: high glucose condition (28 mmol/L); A: ALA; LY: LY294002 (10 µmol/L); W: wortmannin (100 nmol/L). Data are presented as mean±SEM of 3 independent experiments performed in triplicate. ^# #^ *P*<0.01 *vs.* NG. ^*^ *P*<0.05,^ **^ *P*<0.01 *vs.* HG. ^ηη^ *P*<0.01 *vs.* HG+A.

Dietary ALA can be converted to long-chain n-3 PUFAs in humans and may reproduce some of the beneficial effects of EPA and DHA on cardiovascular disease risk factors. In order to determine whether ALA has a direct anti-adhesive and endothelial protective effects against high glucose injury, endothelial cells were cultured and exposed to high glucose. As is shown in Figure 3A, stimulation with 28 mmol/L high glucose (HG group) for 48 hours caused an about three-fold increase in neutrophils adhesion to HUVECs compared with normal glucose group (NG group, 5.5 mmol/L). Pretreatment with different concentrations (10, 50, 100 µmol/L) of ALA for 18 hours attenuated high glucose-induced neutrophils adhesion to HUVECs, with a maximal effect at 50 µmol/L (13.67±1.35% *vs.* 21.99±0.84% in HG group, *P<*0.01). In contrast, when increasing ALA’s concentration to 200 µmol/L, neutrophils adherence (28.06±2.83%) was even higher than that of HG group (*P<*0.05). Based on these observations, further experiments were carried out using ALA at the concentration of 50 µmol/L.

### ALA Reduced High Glucose-mediated CAMs Expression in HUVECs

**Figure 4:**
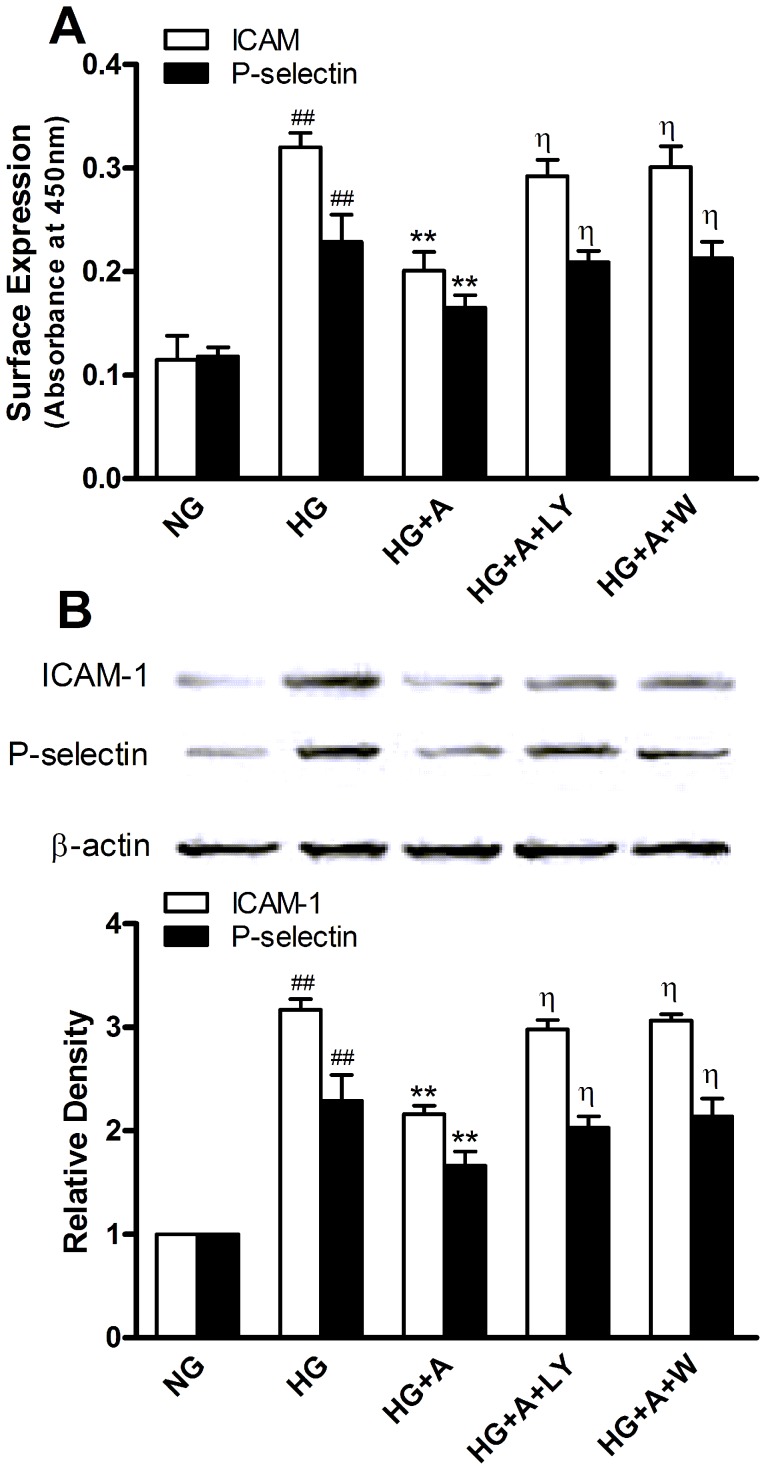
Surface (A) and protein expression (B) of ICAM-1 and P-selectin in HUVECs. NG: normal glucose condition (5.5 mmol/L); HG: high glucose condition (28 mmol/L); A: ALA; LY: LY294002 (10 µmol/L); W: wortmannin (100 nmol/L). Values are presented as mean±SEM of 3 independent experiments performed in triplicate (Figure A). Data obtained from quantitative densitometry were presented as mean±SEM of 3 independent experiments (Figure B).^ # #^ *P*<0.01 *vs.* NG. **P<0.01 *vs.* HG. ^η^ *P*<0.05 *vs.* HG+A.

Adhesion of neutrophils to endothelial cells is largely mediated by adhesion molecules including P-selectin and ICAM-1. So we further investigated the effects of ALA on the surface expression (Figure 4A) of P-selectin and ICAM-1 and their protein expression (Figure 4B) in HUVECs stimulated by high glucose. Both the surface expression of these two molecules and their protein expression in unstimulated cells (NG group) were very low. After endothelial cells were exposed to high glucose (28 mmol/L) for 48 hours, surface and protein expression of P-selectin and ICAM-1 significantly increased (both *P<*0.01 *vs.* NG group). Pretreatment with 50 µmol/L ALA significantly inhibited high glucose-induced P-selectin (0.17±0.01 *vs.* 0.23±0.03 in HG group, *P<*0.01) and ICAM-1 (0.20±0.01 *vs.* 0.32±0.02 in HG group, *P<*0.01) expression in HUVECs.

### ALA Inhibited High Glucose-induced Neutrophil Adhesion and CAM Expression in HUVECs via the PI3K/Akt Pathway

**Figure 5:**
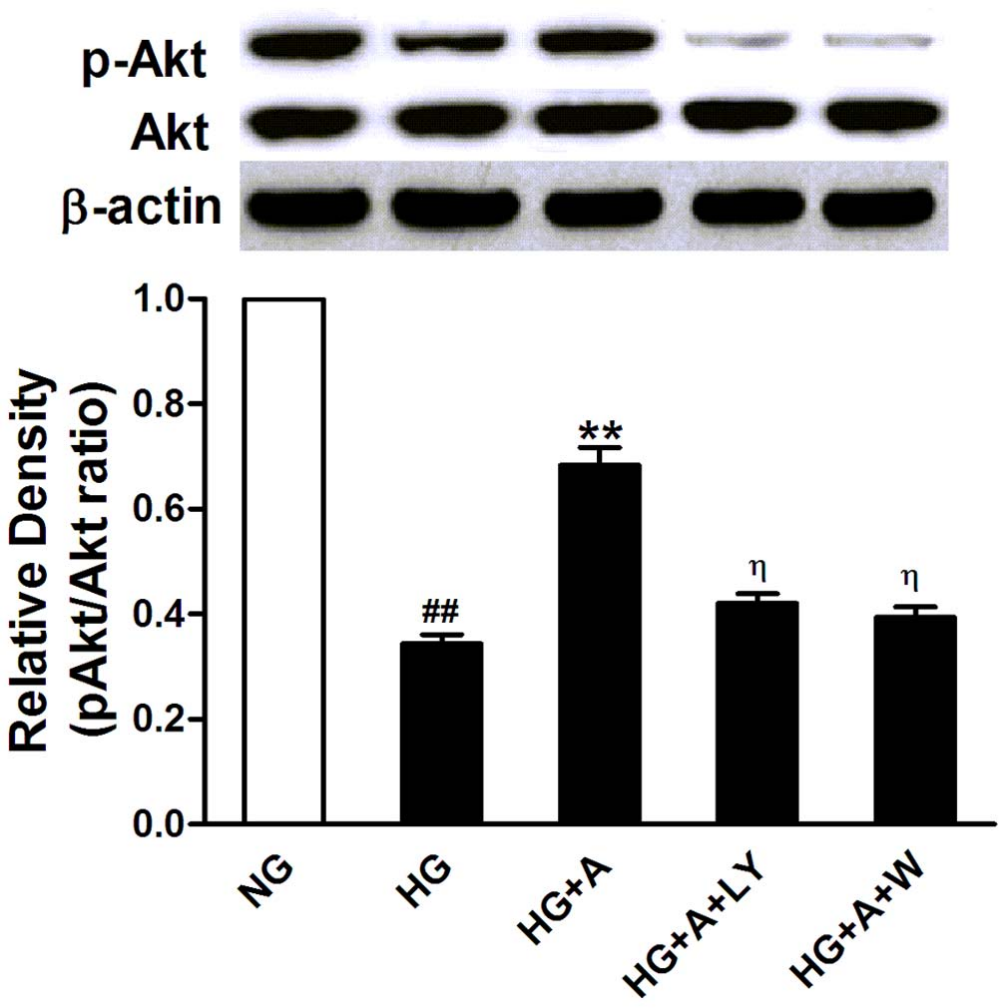
Phosphorylation of Akt in HUVECs exposed to high glucose with different treatments. NG: normal glucose condition (5.5 mmol/L); HG: high glucose condition (28 mmol/L); A: ALA; LY: LY294002 (10 µmol/L); W: wortmannin (100 nmol/L). Data obtained from quantitative densitometry were presented as mean±SEM of 3 independent experiments. ^#^ *P*<0.05 *vs.* NG. *P<0.05 *vs.* HG. ^η^ *P*<0.05 *vs.* HG+A.

To explore the potential mechanism for anti-adhesive property of ALA in HUVECs, we examined the effects of ALA on Akt expression and phosphorylation. Though high glucose did not affect Akt expression in HUVECs, it significantly reduced Akt phosphorylation (*P<*0.05 *vs.* NG group). Preincubation of HUVECs with ALA caused a significant increase in Akt phosphorylation compared with HG group, which was abolished by the PI3K inhibitors LY294002 (10 µmol/L) and wortmannin (100 nmol/L) (Figure 5). ALA had no effects on Akt protein expression. Interestingly, both LY294002 and wortmannin blocked the inhibition of ALA (50 µmol/L) on adhesion molecules expression (Figure 4) and adherence of neutrophils to HUVECs (Figure 3B) exposed to high glucose, suggesting that ALA may exert an anti-adhesive effect in HUVECs exposed to high glucose via the PI3K/Akt pathway.

## Discussion

Several important observations were made in the present study. First, our data indicated that *in vivo* treatment with ALA inhibited endothelial inflammation, as manifested by decreased circulating levels of endothelial cell adhesion molecules, and improved endothelial function in STZ-induced diabetic rats. Second, we demonstrated for the first time that *in vitro* treatment with ALA acted directly on endothelial cells to attenuate high glucose-induced endothelial adhesion molecules expression and neutrophils adhesion via a PI3K/Akt mechanism, suggesting that the PI3K/Akt pathway contributes to the anti-adhesive effect of ALA against high glucose injury.

Impairment of endothelium-dependent vasodilatation has been described in diabetic patients [25], [26] and animal models of diabetes and spontaneous hypertension [18], [24], [27]. It has been reported there is a close relationship between endothelial dysfunction and metabolic control in STZ-induced diabetic rats [27]. In the present study, a reduction in endothelium-dependent vasodilatation has been observed in isolated aortic rings and treatment with ALA improved endothelium-dependent vasodilatation significantly in STZ-induced diabetic rats, indicating the vasculoprotective effect of ALA in diabetes. Vascular inflammation and endothelial activation play important roles in the pathogenesis of diabetic vascular complications [20]. Expression of endothelial adhesion molecules promotes the adherence and transmigration of leukocytes into the subendothelial space, eventually leading to endothelial structural change and endothelial dysfunction. Endothelial activation can be assessed by measuring circulating levels of endothelial soluble adhesion molecules such as sP-selectin and sICAM-1 [4], [20]. Our results showed that STZ-induced diabetic rats had significantly higher levels of sP-selectin and sICAM-1 whereas *in vivo* treatment with ALA decreased both sP-selectin and sICAM-1 in diabetic rats. Furthermore, our *in vitro* experiment demonstrated that ALA (50 µmol/L) suppressed high glucose-induced expressions of ICAM-1 and P-selectin in HUVECs, which resulted in reduced neutrophils adherence to HUVECs. Collectively, these data suggest that ALA exerts a direct anti-adhesive effect on endothelial cells under high glucose condition and thus inhibits endothelial activation in diabetes. Our findings, together with recent clinical data about ALA in dyslipidemia patients [11], [12], imply an anti-inflammatory effect of ALA in metabolic disorders.

In the present study, lower concentrations (10, 50, 100 µmol/L) of ALA inhibited high glucose-mediated neutrophils adherence to cultured HUVECs whereas a higher concentration (200 µmol/L) of ALA induced more neutrophils adhesion to HUVECs than high glucose alone. Furthermore, our data indicated that ALA (50 µmol/L) suppressed high glucose-induced expression of ICAM-1 and P-selectin in HUVECs, both on cell surface and on protein level. Zhao *et al* also reported that ALA exerted the anti-inflammatory effect in THP-1 cells. [28]. Their study showed that ALA, at doses ranging from 0 to 100 µmol/L, reduced the lipopolysaccharide (LPS)-stimulated production of interleukin (IL)-6, IL-1β and tumor necrosis factor (TNF)-α by human monocytic THP-1 cells in a dose-dependent manner. The seeming discrepancy of optimum dose with maximum anti-inflammation effect between our results and those of Zhao *et al.* may be due to differences in the cell lines and stimulus selected. In our study, a pro-inflammation effect was observed when the concentration of ALA was increased to 200 µmol/L; while Zhao *et al.* only observed the effect of ALA with a max dose of 100 µmol/L. We supposed that the pro-inflammatory effect of high concentration of ALA to HUVECs may be ascribed to the interactions between fatty acids and glucose, which can cause oxidative stress [29]. However, the exact mechanisms need further study.

In contrast to the findings from the present study, Toborek *et al* reported that exposure of endothelial cells to ALA stimulated the development of pro-inflammatory environment within vascular endothelium [30]. The opposite results may be relevant to the different stimulus selected: the stimulus selected in Toborek study is TNF-α, a much stronger inflammatory stimulus than high glucose. Another thing we should emphasize is that in the study conducted by Toborek, the effects of fatty acid on inflammatory responses were compared with a control that had no inflammatory stimulus, whereas the present study evaluated the ALA effects on high glucose-induced inflammatory response. In addition, as described by Toborek *et al*, when compared with the TNF-α treatment, cells treated with ALA had a lesser activation on NF-κB, indicating a diminished inflammatory response. Based on this interpretation, the findings from these two studied are similar.

Recent evidence has revealed that PI3K/Akt pathway plays a role in the cellular inflammatory response [31]. The regulatory role of the PI3K/Akt signal cascade in inflammatory process appears to be cell- and ligand-specific. Our data showed that ALA treatment increased Akt phosphorylation, attenuated P-selectin and ICAM-1 expression and inhibited neutrophils adhesion in HUVECs exposed to high glucose, all of which were blocked by the PI3K inhibitor LY294002 and wortmannin. These results suggest that the PI3K/Akt pathway contributes to the anti-inflammatory effect of ALA in high glucose-induced HUVECs. Chen *et al.* also reported that DHA and EPA attenuate ox-LDL-induced expression of adhesion molecules and the adhesion of monocytes to human coronary artery endothelial cells by modulation of Akt activation [15]. However, the downstream molecules of the PI3K/Akt pathway through which ALA exerts its anti-inflammatory effect have not been fully examined.

Taken together, our data indicated that ALA treatment improved endothelial function and inhibited endothelial activation in STZ-induced diabetic rats. Of note that ALA acted directly on endothelial cells to attenuate high glucose-induced endothelial adhesion molecules expression and neutrophils adhesion via increasing Akt phosphorylation, suggesting that the anti-adhesive effect of ALA against high glucose injury may partially be mediated by the PI3K/Akt pathway. These findings provide potential benefits of ensuring adequate ALA intake as a strategy to prevent against diabetic vascular complications. We believe that incorporating food sources of ALA in the diet, such as walnuts, walnut oil, and flaxseed oil, is important for the evolving dietary strategies to reduce cardiovascular disease risk.
